# Low density neutrophils are increased in patients with Behçet’s disease but do not explain differences in neutrophil function

**DOI:** 10.1186/s12950-022-00302-1

**Published:** 2022-03-31

**Authors:** Mariam Murad, Liying Low, Matthew Davidson, Philip I. Murray, Saaeha Rauz, Graham R. Wallace

**Affiliations:** 1 Academic Unit of Ophthalmology, Institute of Inflammation and Ageing, University of Birmingham, Edgbaston, B15 2WD UK; 2 Birmingham & Midland Eye Centre, Sandwell and West Birmingham Hospitals NHS Trust, Birmingham, UK

**Keywords:** Low density neutrophils, Behcet’s disease

## Abstract

**Background:**

Behçet’s disease (BD) is a multisystem autoinflammatory disease characterised by mucosal ulceration, ocular, neural, joint and skin inflammation. The cause of BD is not known but there is a strong genetic association with HLA-B*51, IL10 and IL23R. Neutrophils are a first line of defence against invading pathogens and have been described as activated in patients with BD. Neutrophils can now be separated into different subsets, such as low density (LDN) and normal density (NDN) that have diverse functional roles. We wished to address neutrophil heterogeneity in patients with BD.

**Methods:**

Peripheral blood neutrophils were obtained from 32 BD patients and 37 healthy aged-matched controls. Percoll isolation was used to isolate all neutrophils, while Ficol-Hypaque was used to obtain LDN and NDN. Phagocytic capacity and production of reactive oxygen species (ROS), and neutrophil extracellular traps (NET) stimulated with phorbol 12-myristate 13-acetate (PMA) and *Escherichia coli* (*E.coli*) were assessed in both groups.

**Results:**

We have demonstrated reduced phagocytic capacity and ROS production but greater NET production by total neutrophils stimulated with PMA or *E.coli* from BD patients in comparison with healthy controls. Patients with BD had elevated numbers of LDN and lower number of NDN compared with healthy controls. However, both neutrophil subsets showed the same reduced ROS production and phagocytic function as total neutrophils in both groups.

**Conclusion:**

Our novel findings indicate that the neutrophil population in BD is heterogeneous and the increased number of LDN in combination with greater NET production may contribute to the inflammatory response and pathogenesis.

**Supplementary Information:**

The online version contains supplementary material available at 10.1186/s12950-022-00302-1.

## Introduction

Behçet’s disease (BD) is a multisystemic inflammatory disorder characterised by recurrent oral ulcers, genital ulcers, intraocular and neural inflammation skin lesions and thrombotic vascular events [1]. The cause of BD remains unknown despite a strong association with HLA-B*51, *IL10 and IL23RIl12RB* [2] The prevalence rate of BD in the UK is 14.61 (95% CI 13.35–15.88) per 100,000 population [3]. Between 50 and 90% of BD patients have intraocular involvement manifestations, such as recurrent, non-granulomatous panuveitis, hypopyon and retinal vasculitis. Posterior segment inflammation of the eye also involves occlusion of retinal veins and later occlusion of the retinal arterial circulation that can lead to irreversible blindness [4]..

Neutrophils are a major group of the immune cell population that are present in a resting state in the circulation of healthy individuals and play a dynamic role within an inflammatory response. Neutrophils are phagocytic cells that ingest infectious agents, such as bacteria and fungi, that are destroyed by the production of reactive oxygen and nitrogen species (ROS and NOS) known as oxidative burst, with the release of neutrophil granule components and the production of elastase [5]. The build-up of oxidative stress and the production of oxygen radicals may cause DNA damage, oxidation of lipids, lipoproteins and proteins and can be linked to mutations in immunoglobulins that have been implicated in the formation of inflammatory diseases [6]. Neutrophil extracellular traps (NET) are web-like structures composed of cytosolic and granule proteins of decondensed chromatin, produced to protect against infection by pathogens [7]. NET proteins are derived from primary granules (neutrophil elastase and myeloperoxidase), secondary granules (lactoferrin and pentraxin 3) and tertiary granules (matrix metalloproteinase). NET formed by activated neutrophils trap bacteria and other pathogens leading to their destruction [8].

Neutrophil hyperfunction, in particular increased neutrophil chemotaxis, has been linked with BD for over 40 years [9]. Neutrophils have been reported in biopsies of active oral and genital ulcers, the skin lesions of erythema nodosum and the skin pathergy test [10, 11]. Neutrophils from HLA-B*51-positive patients showed an increase in the chemotactic response toward several stimuli suggesting that the HLA region can exert a regulatory control on PMN functions [12, 13]. γδ T cells and monocytes activated via Toll-like receptor-2 (TLR2) have been implicated in releasing neutrophil stimulating molecules that induce chemotaxis in neutrophils from patients with BD and healthy controls in response to several stimuli [14]. Neutrophils from BD patients display elevated superoxide production and increased lysosymal enzyme production in response to different stimuli [15, 16]. Recent studies have shown increased NET production by neutrophils from patients with BD [17, 18]. Increased expression of CD11b possibly as a result of the interaction of neutrophils with activated platelets, enhanced platelet-neutrophil aggregate formation [19].. The neutrophil-lymphocyte ratio was significantly higher in patients with active BD, although not significantly different between patients with or without thrombosis [20]. Certain patients with BD can be successfully treated with colchicine which is a neutrophil inhibitor [21, 22].

Neutrophils were considered to be a homogenous population of differentiated cells with a distinct and conserved function. However, increasing evidence has demonstrated a phenotypic heterogeneity and functional flexibility of the neutrophil population. Low density neutrophils (LDN) are banded and appear as myelocyte-like cells [23]. LDN are a heterogenous population including immature cells based on morphology and gene expression, and mature cells based on surface markers (CD66b, CD11b). LDN can be subdivided into those with a pro-inflammatory phenotype and function and those who have been classified as granulocytic myeloid derived suppressive (G-MDSC) with an immunosuppressive phenotype and function [24, 25]. Normal density neutrophils (NDN) separate with the red-blood cell layer in a Ficoll gradient and are described as a homogenous population of resting neutrophils [24].

In this paper we have assessed neutrophil phenotype and function in patients with BD. The results show increased LDN and NET production but decreased production of ROS and phagocytosis in disease cohort compared to controls.

## Material and methods

### Patient samples

Peripheral blood was obtained from 32 patients with BD attending the Birmingham National Centre of Excellence for BD. All patients fulfilled the 1990 International Study Group criteria for BD [26]. Age matched healthy control samples were obtained from University of Birmingham laboratory staff (*n* = 37). Informed written consent was obtained. The research followed the tenets of the Declaration of Helsinki and was approved by the NHS Research Ethics Committee (LREC ref.: 08/H1206/165).

### Total neutrophil isolation

Peripheral venous blood samples were collected in a micro vacutainer containing heparin and processed within 4 h. Under sterile conditions the samples were dispensed into a 50 ml Falcon® tubes and 2% dextran added at a ratio of 1 ml dextran: 6 ml blood and the tubes inverted 5 times. The Falcon® tubes with a loosened lid were left in the hood for 30–40 min, for yellow coloured buffy coat to appear. 5mls of 56%, Percoll was added into a 15 ml Falcon® tube, then 2.5 ml of 80% Percoll was underneath to form a gradient. Buffy coats were added to the gradients and centrifuged at 1100 rpm for 20 min at room temperature with one acceleration and zero de-acceleration. Two distinct layers of cells on the gradients were observed. The top layer represented the peripheral blood mononuclear cells (PBMC) was discarded. The second layer containing neutrophils were collected and placed into a new 15 ml Falcon® tube containing RPMI 1640 medium+ foetal calf serum (10% FCS) + L-Glutamine–Penicillin–Streptomycin solution (1% L-GPS) (completed medium; CM) and centrifuged or 10 min at 1600 rpm at room temperature with one acceleration and zero de-acceleration. Total neutrophils were isolated (purified) using the EasySep Human Neutrophil Enrichment Kit, according to the manufacturer’s instructions, and re-suspended in CM.

### Low and normal density neutrophil isolation

Blood samples were collected in a micro vacutainer containing heparin and processed within 4 h. The Ficoll-Hypaque (FH) solution under sterile conditions was placed into a 50 ml Falcon® tube in the ratio of 2 ml of FH:1 ml of blood. The diluted blood was slowly layered over the FH solution and centrifuged for 40 min at 400 g at 22 °C with 0 break. After centrifugation the LDN, located at the interface between plasma and FH layers (PBMC), were removed. The cells were transferred to a 15 ml Falcon® tube containing 10 ml of PBS and centrifuged for 10 min at 400 g at 4 °C. The supernatant was discarded, the wash repeated and the cells were purified using the EasySep Human Neutrophil Enrichment Kit, according to manufactures instructions. NDN, granulocyte layer, located above the FH were removed and 100 μl of the cell suspension was transferred to 15 ml Falcon® tube. The cell suspension was lysed with red blood lysis buffer in the ratio of 100ul:1 ml, vortexed and incubated in the dark for 20 min. The cells were centrifuged at 250 g for 5 min at room temperature, the supernatant was discarded and NDN were also purified using the EasySep Kit. Both LDN and NDN percetages were identified by morphology on cytospin; subpopulation/all cells × 100.

### Cell viability and purity

Viability of neutrophil populations was assessed by Trypan Blue staining. To determine purity cells were re-suspended in 50 μl of CM, slides constructed and 50 μl of the sample was added into the funnel and the samples were spun in a cytospin centrifuge at 300 rpm for 10 min. Post spinning, the slide was left to air dry for 30 min, dipped into cold methanol for 30 s and left to air dry for 10 min. A small drop of diluted Giemsa (1/20) was added onto the cells and the slide was further left for 30 min. The slide was rinsed thoroughly with distilled water and left to air dry for 1 h. The slide was visualised under a light microscope using X200 magnification. (Supplementary Fig. 1).

### Neutrophil phagocytosis and oxidative burst in total and neutrophil subpopulations

Neutrophil phagocytosis and oxidative burst activity in whole blood samples (total neutrophils), LDN and NDN were quantified using Phagotest and Phagoburst kits (Glycotope Biotechnology). Briefly, for Phagotest cells were aliquoted (100 μl) into pre-labelled Fluorescence-activated cell sorting (FACS) (BD) tubes.Twenty microliter of fluorescein isothiocyanate (FITC)-labelled *Escherichia coli* (*E.coli*) (1–2 × 10^9^ per ml) was added to all control and test samples. Control samples were incubated on ice and the test samples were vortexed for 5 s and were further incubated at 37 °C for 10 min, then placed on ice. Trypan blue (100 μl) was added (on ice) to all samples (controls and test) and vortexed for 5 s. The samples were washed with 3 mls of washing buffer and vortexed, and then centrifuged at 4 °C for 6 min at 250 x g. The supernatant was removed without disturbing the pellet formed.

For Phagoburst aliquoted (100 μl) into pre-labelled FACS tubes. A control sample was set up by adding 20 μl of the wash buffer for each different blood sample, and 20 μl of non-labelled *E.coli* was added to test samples for oxidative burst analysis. The control samples were placed on ice and the test samples were vortexed for 5 s, and then incubated at 37 °C for 10 min. After incubation, the tubes were returned to ice and 20 μl of the Phagoburst substrate (dihydrorhodamine 123) was added all tubes. The tubes were flicked to re-suspend the cells and incubated at 37 °C for 10 min.

Lysing solution (2 mls) was added to all Phagotest and Phagoburst samples (control and test samples) vortexed for 5 s and incubated for 20 min in the dark at room temperature, then centrifuged for 5 min at 250 g at 4 °C. The resultant supernatants were removed and the cells were re-suspended in 100 μl of the residual volume, washed with 3mls of wash buffer and centrifuged at 250 x g for 5 min at 4 °C. The resultant supernatant was discarded and the cells were re-suspended in 100 μl of the residual volume. All samples were stained with 200 μl of DNA staining solution, vortexed for 5 min and incubated on ice for 10 min. Samples were analysed using Accuri flow cytometer within 1 h of preparation and 10,000–15,000 neutrophils were counted per sample (Glycotope Biotechnology). The percentage of neutrophils undergoing phagocytosis or producing reactive oxygen metabolites and accompanying mean fluorescent intensity was measured.

### Generation of cell free DNA (cfDNA)

In this paper cfDNA will be used as a marker of NET production. Isolated neutrophils (2 × 10^5^ cells/ml) were seeded (200 μl) into wells of clear flat bottomed 96 well plates and stimulated with 25 nM PMA (diluted from stock solutions with CM for 3 h at 37 °C and 5% CO_2_ atmosphere. After incubation the supernatant was removed, transferred into 600ul Eppendorf tube and centrifuged at 2200 g for 10 min at 4 °C. 100ul of cell free supernatant was removed and placed into black 96 well plates and incubated with 1uM SYTOX Green Dye for 10 min in the dark at room temperature. The fluorescence was measured using BioTek Synergy 2 fluorometric plate reader with excitation at 485 nm and emission at 528 nm. All samples were analysed in duplicate. A calibration step was performed using the cell free supernatant from unstimulated neutrophils and buffer controls were analysed in duplicate. λDNA (0.3 μg/μl was diluted 1:20 in PBS) was used to calibrate the samples with a standard curve ranging from 0 to 1000 ng/ml. The top standard was serially diluted 7 times with a final dilution of 1:15 with 1uM SYTOX Green Dye. The same assay was followed using opsonised *E.coli* (1–2 × 10^9^ bacteria per ml).

### Visualisation of cfDNA

Purified neutrophils (2 × 10^5^ cells /ml) were re-suspended in 2 ml of CM and seeded onto 8 well chamber slides and incubated for 30 min at 37 °C in a 5% CO_2_ atmosphere to allow cell adherence. After 30 min incubation neutrophils were stimulated with 25 nM PMA for 3 h at 37 °C in a 5% CO_2._ All agonists were prepared using the stock solutions in CM which also served as a buffer control. Post-stimulation, cells were fixed with 4% paraformaldehyde (PFA) (200 μl) and incubated for 30 min at 37 °C in 5% CO_2._ Post fixation, the slides were washed in sterile phosphate-buffered saline (PBS) 3 times, once with 0.1% Triton X-100 for 1 min and finally a 5-min wash in sterile PBS, all at room temperature. Cells were incubated (5 min) with 1 μM SYTOX green Dye at room temperature followed by a 5-min wash in sterile PBS at room temperature. The slides were then mounted in fluoromount medium and were imaged using a LEICA DMI 6000B microscope at × 10, × 20 and × 40 objective. The same assay was followed using opsonised *E.coli* (1–2 × 10^9^ bacteria per ml).

### Statistical analysis

The data was statistically analysed using the Kruskal-Wallis test and Dunn’s Multiple Comparison Test (Post-Hoc) for a comparison between 3 different groups, and a Mann-Whitney U test was performed for a comparison between 2 different groups using GraphPad Prism version 5.03. All *p* values were considered significant at *p* < 0.05. The ratio of NET was calculated by using the following formula;

$$\frac{\mathrm{Stimulated}\ \left(\mathrm{PMA}/\mathrm{E}.\mathrm{coli}\right)}{\mathrm{Unstimulated}\;\left(\mathrm{PMA}/\mathrm{E}.\mathrm{coli}\right)\;}$$

## Results

**Fig. 1:**
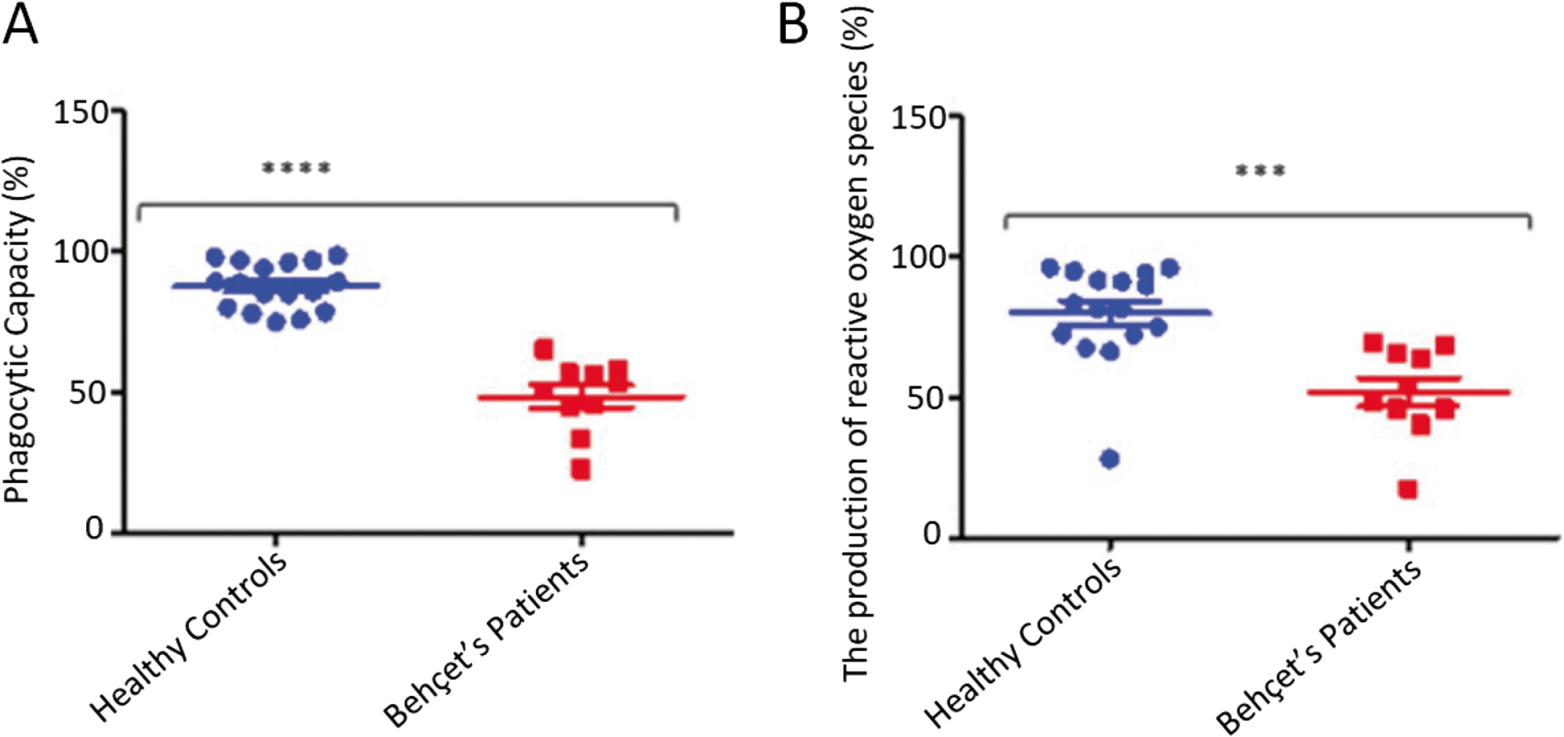
Neutrophils from patients with BD produce less ROS and reduced phagocytosis. **A** ROS production (Phagoburst) in neutrophils of patients with BD (*n* = 10) and healthy controls (*n* = 17). **B** Phagocytic activity (Phagotest), percentage of neutrophils of patients with BD (*n* = 10) and controls (*n* = 16). Analysis by flow cytometry. *P < 0.05, **P < 0.01, ***P < 0.001, *P* < ****0.0001, Mann Whitney U test

**Fig. 2:**
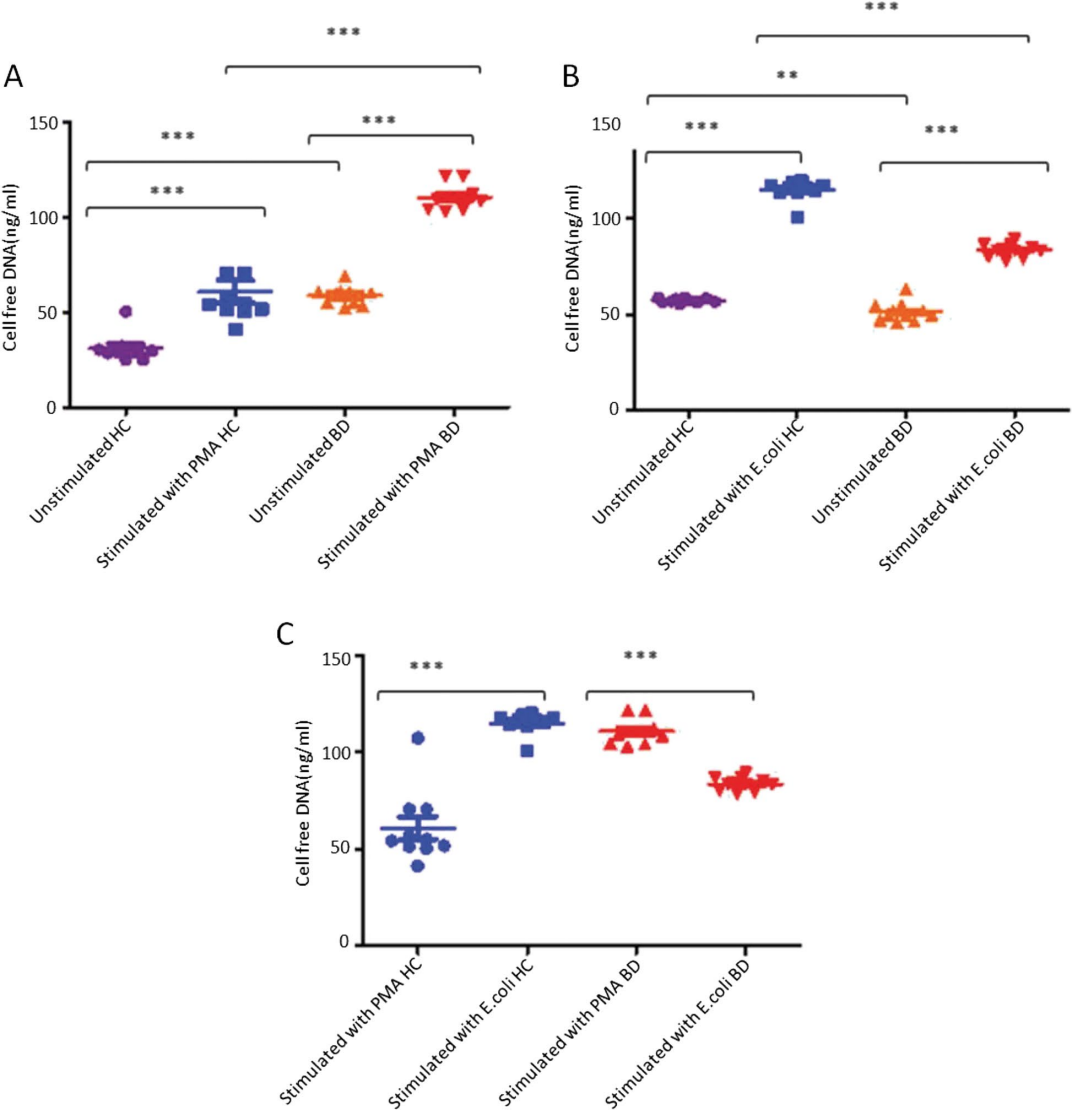
NET production by total neutrophils. **A** The production of NET by PMA stimulated and unstimulated total neutrophils from healthy individuals (*n* = 10) and patients with BD (*n* = 10) was measured by flow cytometric assay. **B** The production of NET in *E.coli* stimulated and unstimulated neutrophils from HC (*n* = 10) and patients with BD (*n* = 11). **C** The production of NETs in stimulated (with PMA and *E.coli*) in neutrophil healthy individuals (*n* = 10) and BD patients (*n* = 1)*P < 0.05, **P < 0.01, ***P < 0.001, *P* < ****0.0001, Mann Whitney U test

Patients with BD (49 ± 4 (%) *n* = 10) had a significantly reduced phagocytic activity of neutrophils compared with healthy individuals (88 ± 2 (%) *n* = 17) (Fig. 1A). Production of ROS was assessed by stimulating neutrophils with opsonised *E.coli* in BD patients (52 ± 5 (%) *n* = 10) produced a reduced amount of reactive oxygen species in comparison to age-matched healthy volunteers (80 ± 4 (%) *n* = 16) (Fig. 1B). Neutrophils were stimulated with PMA to determine release of cell-free DNA. Unstimulated neutrophils from patients with BD (58 ± 2 (ng/ml) *n* = 11) showed significantly greater spontaneous cell free DNA release compared to unstimulated cells from healthy controls (31 ± 2 (ng/ml) *n* = 10). PMA induced significantly greater cfDNA release by neutrophils both from patients with BD (110 ± 2 (ng/ml) vs 58 ± 2 (ng/ml) *n* = 11) and healthy controls (60 ± 5 (ng/ml) vs 31 ± 2 (ng/ml)) compared with unstimulated cells. However, neutrophils from patients with BD (110 ± 2 (ng/ml) vs 60 ± 5 (ng/ml)) responded to stimulus with significantly greater cfDNA release than cells from healthy controls (Fig. 2A).

In a second analysis spontaneous release of cfDNA was greater in unstimulated neutrophils from healthy controls (57 ± 1 (ng/ml)) compared with BD patients (51 ± 5 (ng/ml)). Stimulation with opsonised *E.coli* significantly increased cfDNA release from healthy controls 115 ± 5 (ng/ml) vs 57 ± 1 (ng/ml) and BD patients 84 ± 1 (ng/ml) vs 51.3 ± 1 (ng/ml), however in comparison to PMA stimulation neutrophils from healthy controls produced significantly more than stimulated neutrophils from patients with BD (115 ± 1 (ng/ml) vs 84 ± 1 (ng/ml)) (Fig. 2B). A comparison of PMA and *E.coli* stimulation is shown in Fig. 2C.

**Fig. 3:**
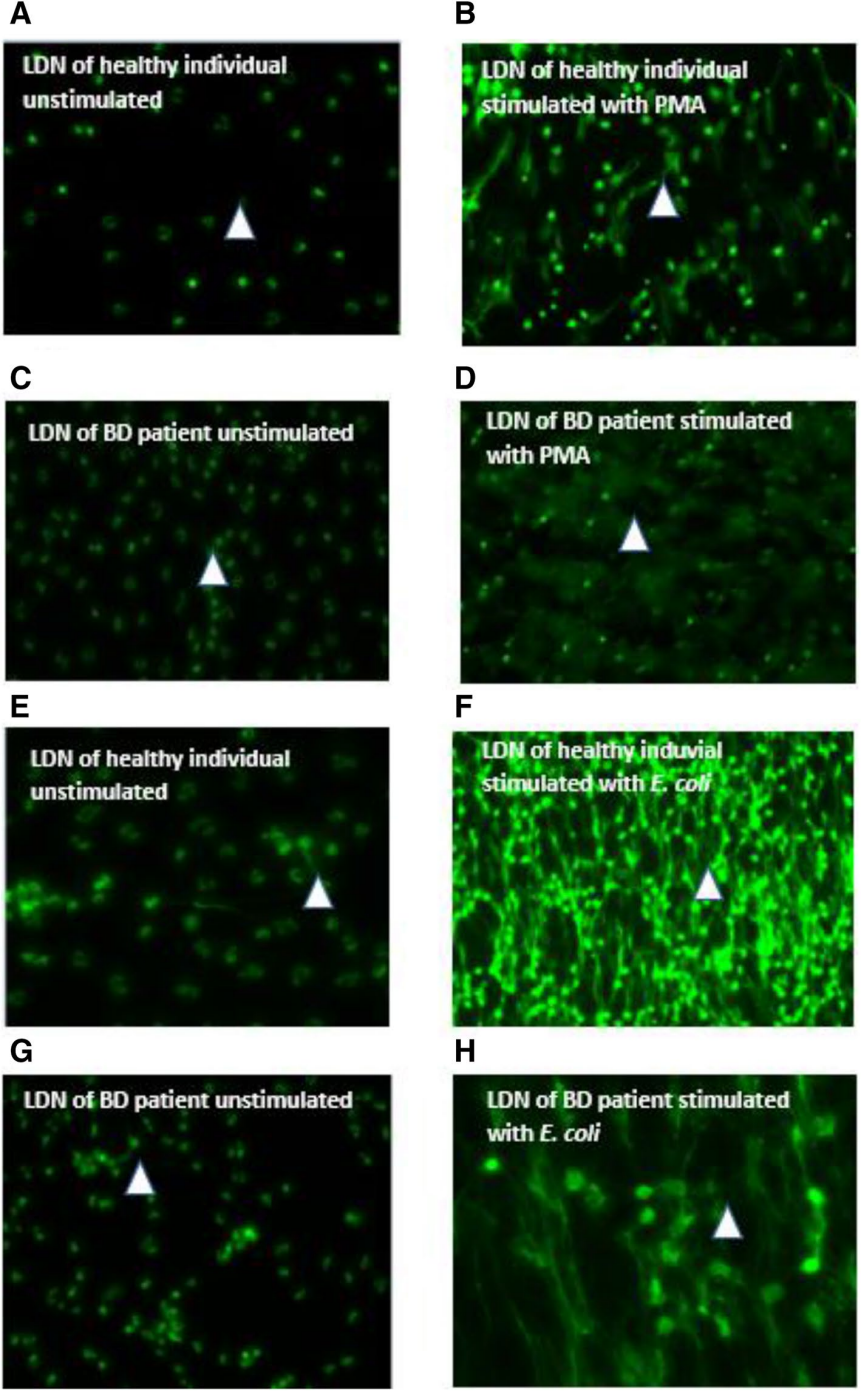
Representative example of NET production - LDN cultures of healthy individuals and BD patients. **A**-**D** The isolated cells cultures were seeded into 8 well chamber slides and stimulated with PMA (25 nM) and opsonised *E.coli* (1–2 × 10^9^ bacteria per ml) (**E**-**H**) and stained with Sytox dye. Images were taken at × 20. Arrow-representing formation of NET (strand of DNA)

In order to visualise NET in stimulated neutrophils with PMA and *E.coli,* Sytox Green was used. The results were comparable to results obtained for the productions of cf. DNA by all neutrophils from patients with BD compared with healthy controls. Figure 3 showed DNA strands i.e. NET formation in neutrophils stimulated with PMA or *E.coli* from patients with BD (Fig. 3B and D).

**Fig. 4:**
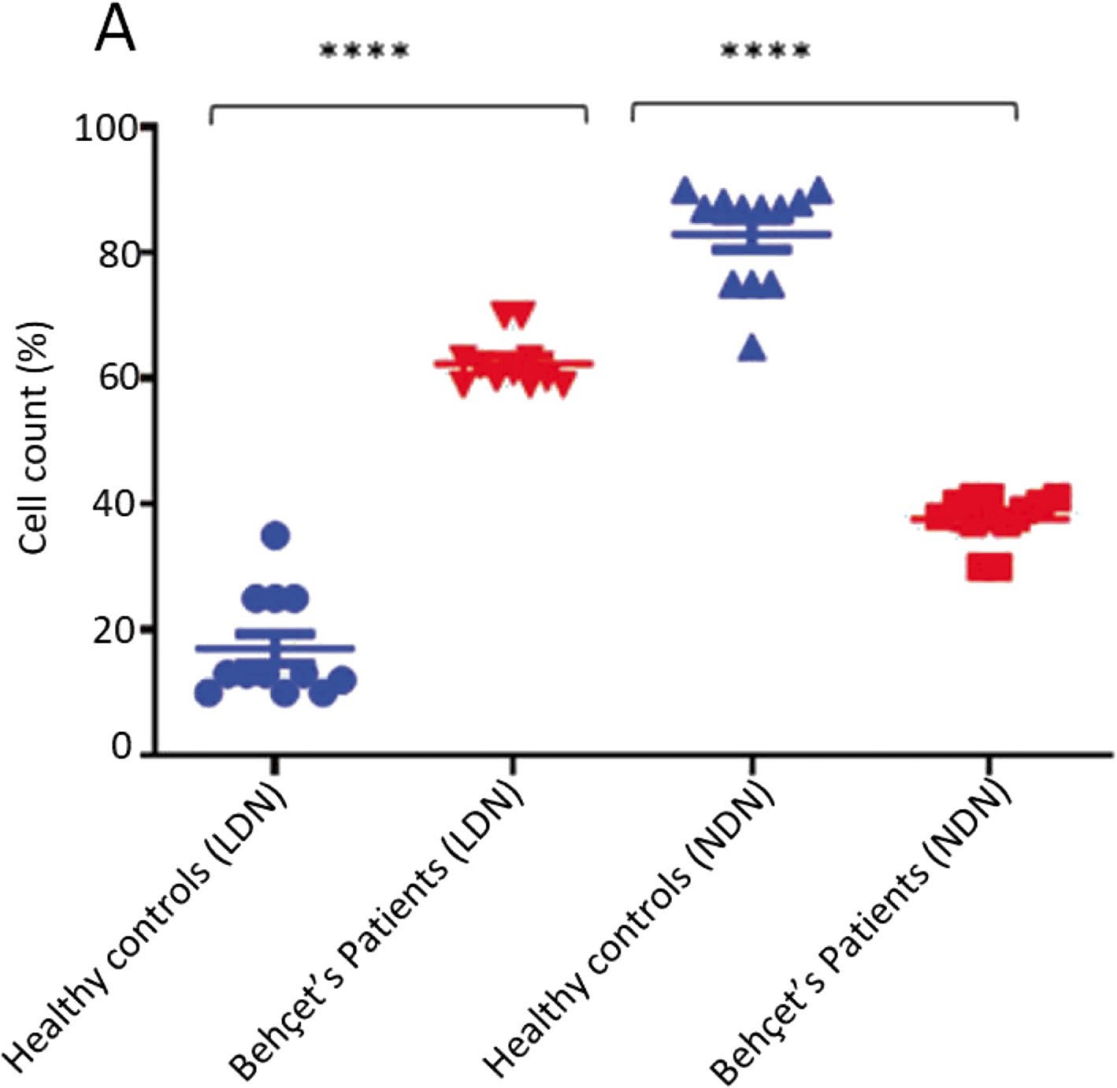
The percentage of LDN and NDN in BD, patients and healthy individuals. **A** The percentage (count) of LDN (*n* = 11) and NDN (*n* = 11) in BD patients in comparison to healthy controls (*n* = 12). The percentage of LDN and NDN was investigated based upon the morphology of the isolated cells using Ficol-hypaque gradient, (subpopulation/all cells × 100). The LDN were isolated from the PBMSC layer and NDN were isolated from the buffy layer on top of red blood cells. ****P < 0.0001, Mann Whitney U test

To address whether the differences in ROS, cfDNA and phagocytosis were due to different subsets of cells, LDN and NDN were prepared on Ficoll gradients. Morphologically, LDN have a curved nucleus with two or fewer nuclear lobes while NDN show mature segmented nuclei. Patients with BD showed an increased LDN (62 ± 4 vs 17 ± 8 (*n* = 11)) and reduced NDN (38 ± 4 vs 83 ± 8 (%) (*n* = 11)) count compared with healthy controls (*n* = 12) (Fig. 4).

**Fig. 5:**
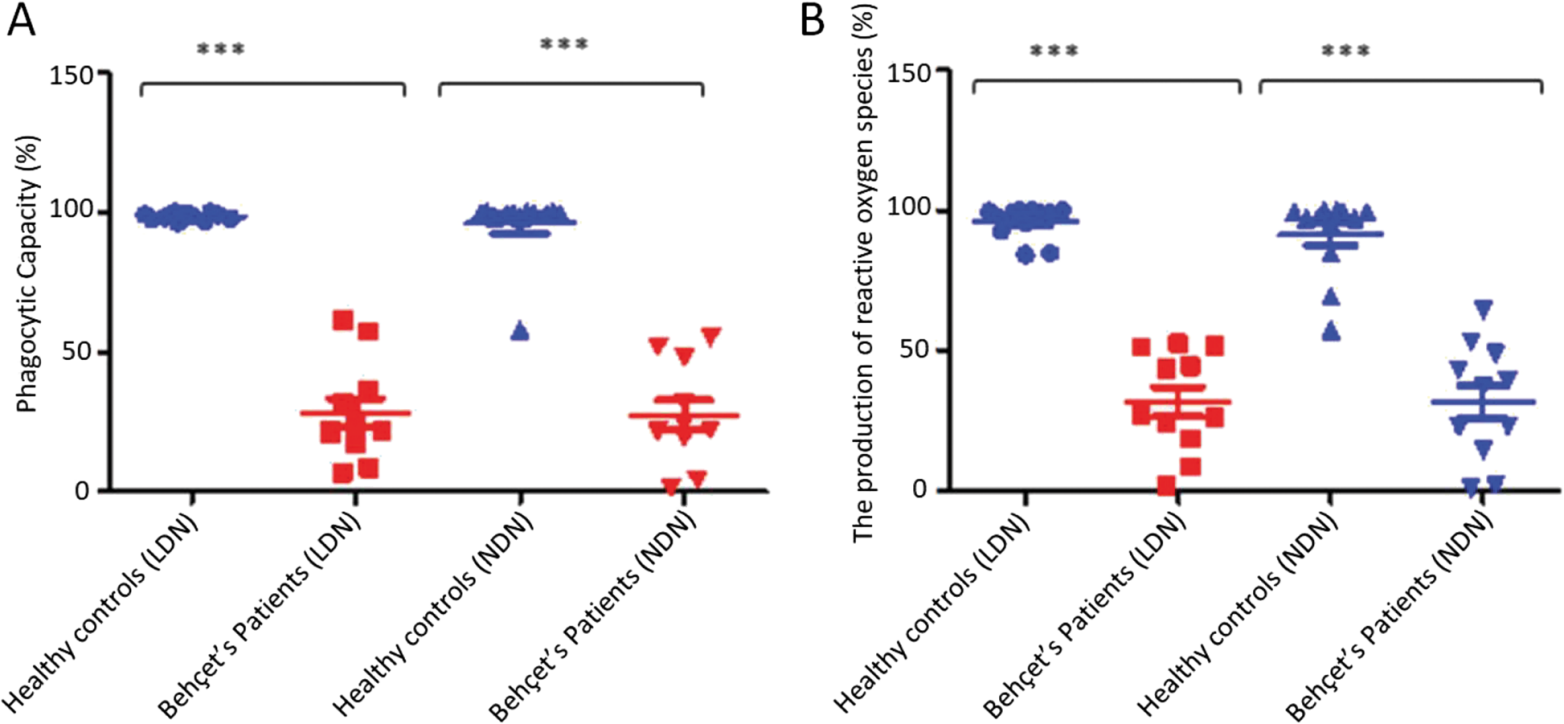
Comparison of phagocytosis and ROS production by LDL and NDN. **A** The phagocytic capacity (Phagotest) of LDN (*n* = 11) and NDN in BD (*n* = 12) patients in comparison to healthy controls (*n* = 12). **B** The production of reactive oxygen species (Phagoburst) by LDN (*n* = 11) and NDN (*n* = 11) in BD patients in comparison to healthy controls (*n* = 12). *P < 0.05, **P < 0.01, ***P < 0.001, ****P < 0.0001, Mann Whitney U test

**Fig. 6:**
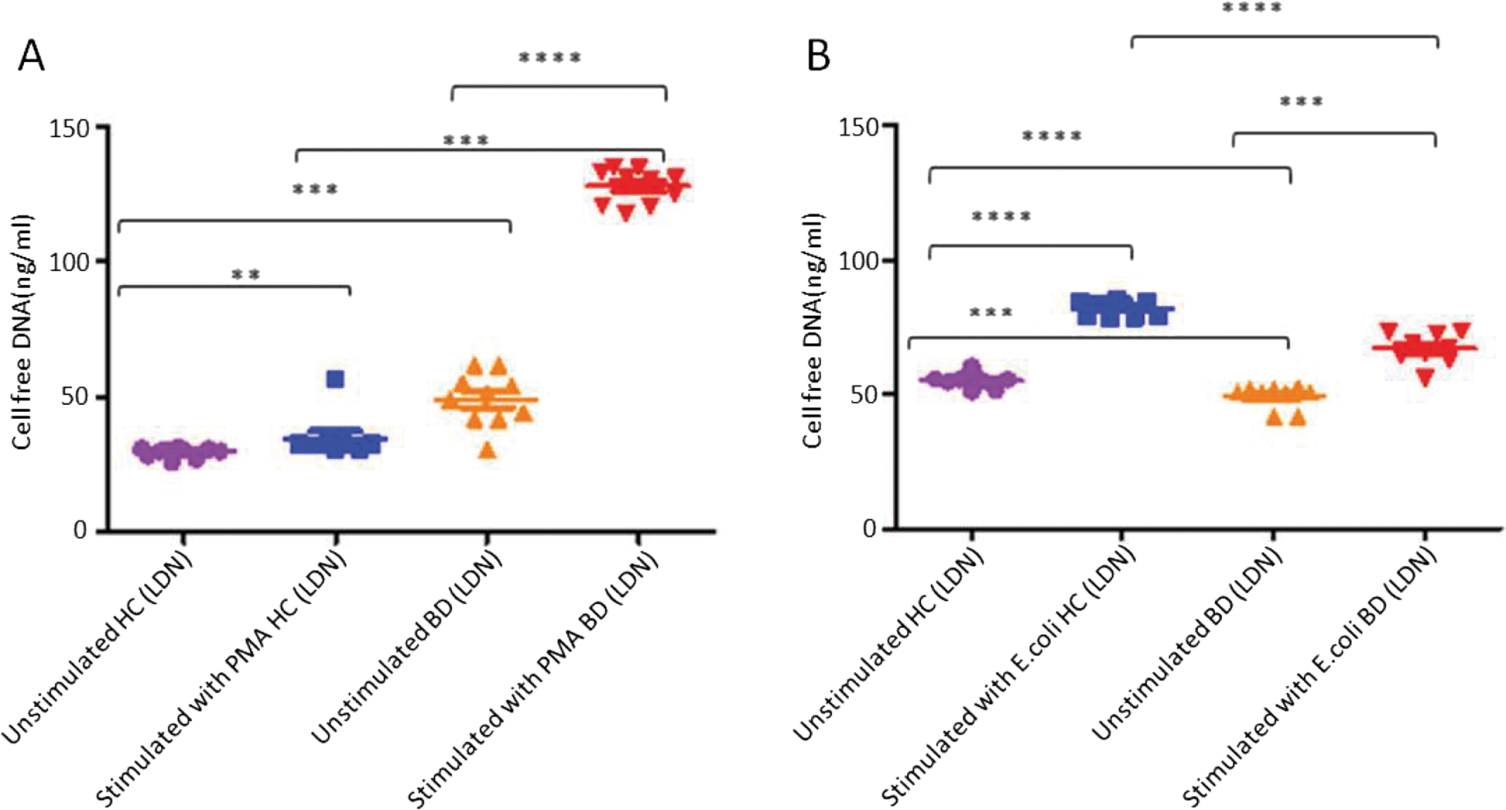
**A** The production of NET stimulated with PMA in LDN from healthy controls (*n* = 10) and BD (*n* = 10) patients, unstimulated healthy controls (*n* = 10) and unstimulated BD patients (*n* = 10). **B** The production of NETs in stimulated with *E.coli* in healthy individuals (*n* = 10) and BD (*n* = 10), unstimulated healthy controls (*n* = 10) and unstimulated BD (*n* = 10), stimulated healthy controls (*n* = 10) and stimulated BD patients (*n* = 10). *P < 0.05, **P < 0.01, ***P < 0.001, ****P < 0.0001

**Fig. 7:**
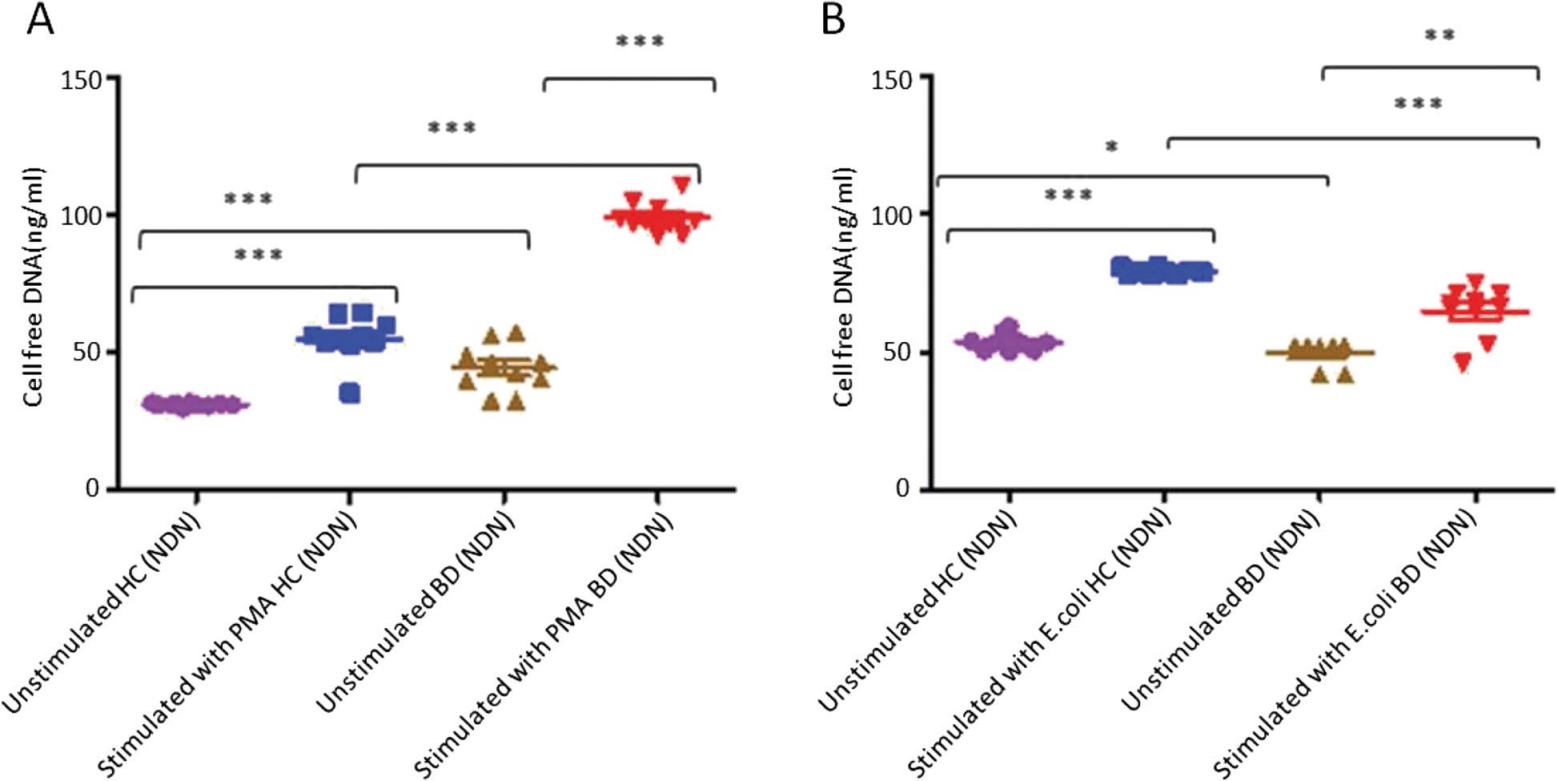
**A** The production of NETs stimulated with PMA in NDN from healthy controls (*n* = 10) and BD (*n* = 10) patients, unstimulated healthy controls (*n* = 10) and unstimulated BD patients (*n* = 10). **B** The production of NETs in NDN stimulated with *E.coli* in healthy individuals (*n* = 10) and BD (*n* = 10), unstimulated healthy controls (*n* = 10) and unstimulated BD (*n* = 10), stimulated healthy controls (*n* = 10) and stimulated BD patients (*n* = 10). *P < 0.05, **P < 0.01, ***P < 0.001, ****P < 0.0001

Whether these populations could account for the differences seen in total neutrophils was considered and LDN and NDN were assessed as above. The results showed a statistically significantly lower phagocytic capacity of both LDN (28 ± 5 (%) (*n* = 12) vs 98 ± 1 (%) (*n* = 11) and NDN (28 ± 5 (*n* = 12) vs 96 ± 3 (%) (*n* = 11) in BD patients compared with healthy controls (Fig. 5A), and a similar decrease in ROS production (32 ± 18 vs 96 ± 3 (%)) (32 ± 6 vs 91 ± 14 (%) (Fig. 5B). Spontaneous cfDNA production by both LDN (49 ± 3 vs 29 ± 1) NDN (44 ± 8 vs 31 ± 1) subsets were significantly greater in patients with BD compared with healthy controls (Figs. 6A and 7A).

Figure 6A shows that cfDNA taken from cultures of PMA stimulated LDN of patients diagnosed with BD was not only significantly higher (128 ± 6 vs 49 ± 10 (%)) than cell free DNA content taken from unstimulated BD cell cultures but was also significantly higher (128 ± 6 vs 35 ± 8 (%)) in comparison to cell free DNA taken from cultures of PMA stimulated neutrophils of healthy individuals (Fig. 6A).

Figure 6A showed that cell free DNA taken from cultures of PMA stimulated LDN of patients diagnosed with BD was not only significantly higher (128 ± 6 vs 49 ± 10) than cell free DNA content taken from unstimulated BD cell cultures, but was also significantly higher (128 ± 6 vs 35 ± 8) in comparison to cell free DNA taken from cultures of PMA stimulated neutrophils of healthy individuals (Fig. 6A). The results showed PMA stimulated LDN of healthy controls was significantly (35 ± 8 vs 30 ± 2) higher in comparison to unstimulated LDN in healthy individuals.

The c/fDNA taken from cultures of PMA stimulated NDN of BD was not only significantly higher (99 ± 6 vs 44 ± 8) than c/fDNA content taken from unstimulated BD neutrophil cultures, but was also significantly higher (99 ± 6 vs 55 ± 8) in comparison to c/fDNA taken from cultures of PMA stimulated neutrophils of healthy individuals (Fig. 7A). The production of c/fDNA from cultures of primed NDN of healthy individuals was also significantly (55 ± 8 vs 31 ± 1) enhanced (Fig. 7A). An increase (44 ± 8 vs 31 ± 1) in spontaneous c/fDNA was observed in NDN of BD patients in contrast to health individuals (Fig. 7A).

While PMA is commonly used to investigate NET production it is a non-physiological stimulus. To address this total neutrophils, NDN and LDN subsets were stimulated with *E.coli* under the same conditions. Stimulation with *E.coli* showed a significantly reduced production of NETs. Figure 6B showed that c/fDNA obtained from cultures of *E.coli* stimulated LDN of BD patients was significantly lower (67 ± 5 vs 82 ± 3) than c/fDNA content taken from *E.coli* stimulated neutrophils of healthy individuals; despite being higher (67 ± 5 vs 49 ± 4) in comparison to unstimulated LDN of BD patients. The results also showed a significant (82 ± 3 vs 55 ± 3) increase in the production of c/fDNA by stimulated LDN in comparison to unstimulated LDN in healthy individuals. The results showed that unstimulated LDN cell cultures produced higher amount (55 ± 3 vs 49 ± 3) of spontaneous c/fDNA in comparison to unstimulated LDN cell cultures of BD patients (Fig. 6B).

Stimulation with *E.coli* showed a significantly reduced production of NET in stimulated (65 ± 9 vs 79 ± 1) neutrophil cell cultures of BD patients in comparison to healthy controls (Fig. 7B). A significant increase (65 ± 9 vs 50 ± 4), of stimulated *E.coli* neutrophil BD cell cultures in comparison to unstimulated was observed. A similar pattern of a significant increase (79 ± 1 vs 54 ± 3) of cell free DNA in stimulated neutrophil cell cultures obtained from healthy controls compared to unstimulated. Figure 7B showed significantly reduced spontaneous c/fDNA in in healthy individuals and BD patients (50 ± 4 vs 53 ± 2).

The results showed the control group (unstimulated cells) for PMA stimulated LDN and NDN of healthy individuals produced less spontaneous c/fDNA in comparison to the control group for *E.coli* stimulated LDN and NDN of healthy individuals in comparison to patients with BD (Fig. 6A, B, 7A, B). However, NET in LDN and NDN with the stimulant *E.coli* was reduced in patients with BD patients in comparison to healthy controls (Figs. 6B, 7B).

## Discussion

Neutrophils have long been implicated in the pathogenesis of BD. Recent advances in neutrophil subsets and functions allowed us to reanalyse this relationship. In our cohort of patients with BD, total neutrophils have reduced phagocytic capacity and ROS production when compared with age matched healthy controls. By comparison, these cells produce more cfDNA in response to stimuli, PMA and *E.coli.* Patients with BD had greater numbers of LDN compared with healthy controls, and both LDN and NDN showed increased cfDNA production on stimulation. Although LDN are increased in patients with BD this does not explain the functional differences between patients and healthy controls.

With regards to ROS production and phagocytosis these results differ from previous reports. Gogus et al. showed significantly higher oxidative burst by neutrophils in patients with BD compared with HC in response to monosodium urate crystals [15]. Perazzio et al. found no significant differences between BD patients and controls with regard to oxidative burst, phagocytic activity, microbicide activity or cytokine production. However, the cells from patients with severe BD exhibited significantly higher oxidative burst activity, both before and after PMA stimulation, compared with cells from patients with mild BD [27]. These differences may be due to the heterogeneity of the patient cohorts, treatment regimens or the effects of the type of stimuli used. In the current studies highly purified neutrophils were used that may have influenced the results. Understanding ROS production is important as neutrophil derived ROS induces carbonylation of fibrinogen leading to increased polymerisation and protection from lysis. Neutrophils from patients with BD were more effective at modifying fibrinogen a process that is involved in clot formation and thrombosis, that may be related to vasculitis in BD [28, 29].

Reduced phagocytic function has been reported during the first 24 h in patients with sepsis was associated with reduced survival. This may represent a neutrophil deactivation state or unresponsiveness due to persistent stimulation by cytokines. Similarly, impaired neutrophil phagocytic function and ROS production was described in patients with anti-neutrophil cytoplasmic antibody associated vasculitis a condition characterised by autoimmune small vessel inflammation [30]. In patients with SLE while ROS production was increased compared with healthy controls, patients with active disease exhibited higher oxidative damage than the inactive group [31]. These findings may be relevant in conditions with a constant inflammatory response with continual stimulation of neutrophils, and re-stimulation of such neutrophils results in a reduced production of functional activity. Although it remains to be clarified, there is no indication that the reduced function of neutrophils from patients with BD is due to the immunosuppressive drugs regimens used to control their condition. One possible mechanism for reduced PMN function in BD could be sleep deprivation.

We have shown for that patients with BD display neutrophil heterogeneity with a high LDN and low NDN count compared with healthy controls. Both LDN and NDN subsets showed reduced ROS production and phagocytic function indicating that difference in numbers of these subsets was not responsible for this phenotype of total neutrophils of patients with BD. These findings were comparable to studies that showed increased levels of LDN in patients with SLE [32]. Moreover, LDN from patients with SLE showed a similar immature phenotype as observed in this study [33]. Recent studies have reported reduced production of ROS and anti-tumour properties by LDN in cancer patients [34]. The presence of LDN may have an important role in the persistent inflammation characteristic and immune response induced in BD patients. A recent review discusses further differences between neutrophil phenotype in different disease with energy metabolism and glycolysis driving ROS and NET production in RA, while decreased redox reactions drive the same in SLE [35].

NET formation has an important role in the development and preservation of autoimmune diseases, organ damage in chronic inflammatory disorders and cancer [36–38]. In our study total neutrophils, LDN and NDN in patients with BD showed increased production of cfDNA compared with healthy controls when stimulated with PMA or *E.coli*. NET can be considered to form part of the host defence system during BD. Inadequate elimination of pathogens due to the reduced phagocytosis and ROS production contributes towards defective killing of bacteria at mucosal surfaces in patients with BD may contribute to the persistent inflammation characteristic of these patients. In a study of Middle Eastern patients with BD PMN from patients released significantly more NET compared to controls. This was linked to expression of higher levels of PAD4, and NET production was reduced using a PAD4 inhibitor. Serum from patients stimulated PMN from healthy controls to produce more NET. NETs were identified around blood vessels in tissue sections from patients with BD linking NET to vasculitis. Moreover, NET from patients with BD decreased proliferation and increased apoptosis when cultured with endothelial cells [17]. Le Joncour et al. described increased serum cfDNA and MPO-DNA complexes in patients with active BD compared with inactive patients and healthy controls, and in patients with vasculitis compared with those without. Plasma from patients with BD induced greater thrombin generation a response correlated with cfDNA and MPO-DNA levels. In support of Safi et al. NETs were identified in areas of vasculitis in biopsy specimens [18]. Patients with both active and inactive BD had Increased levels of sCD40L in their plasma, that induced ROS and NET production in neutrophils, and increased expression of Mac-1 that together could contribute to binding to vasculature [39].

While plasma from patients with BD drives NET production, saliva does not. Saliva of healthy controls, collected in the morning, induced neutrophils to released NET independent of elastase or NADPH. These NET had a greater capacity to kill bacteria and were more resistant to DNAase. By comparison, saliva from patients with aphthous ulcers or BD did not induce NET production due for different reasons. In patients with aphthous ulcers sialyl Lewis X, a molecule driving NET production was lost from salivary mucins. This was not this case in BD as some unknown factor in saliva was inhibiting the response [40]. The possibility that these responses were influenced by altered oral microbiome which is different in patients with aphthous or BD ulceration is intriguing [41, 42]. Likewise the potential influence of other microbiomes at sites of mucosal inflammation in patients with BD on immune responses should be considered.

The results suggested that enhanced production of NET in BD patients may contribute towards the pathology of BD. However, to date most data has utilised imaging cfDNA and it is possible that in inflammatory and necrotic sites this is not all derived from neutrophils [43]. More detailed identification of the components of NET, including histone A3, myeloperoxidase and elastase should be undertaken [44]. Moreover, challenging neutrophils with more physiological stimuli is required. In murine studies PAD4 is not required NET production in all situations and responses to LPS from different sources varies substantially [45, 46]. Although the pathogenesis of BD remains unclear, persistent mucosal ulceration driving a dysfunctional inflammatory response could lead to an increase in neutrophil:lymphocyte ratio, LDN and enhanced NET release and subsequent endothelial activation and vasculitis. While inflammation is treated in patients with BD these results suggest NET production by neutrophils is a novel, attractive therapeutic target in patients with BD [47].

There are several limitations to this study. Firstly, the different analyses were not performed on all the patients or controls due to the requirement to use neutrophils within a few hours of collection. Further studies should analyse phagocytosis, ROS and NET production in the same cells. Secondly patients with BD have different manifestations of the conditions and were on different treatments and this should be addressed in future studies. Finally, as discussed above LDN are not a homogenous, stable population and further investigation of such diversity in BD is needed both in blood and tissue [48].

## Conclusion

This study may indicate towards a dysfunctional phenotype and function displayed by neutrophils in BD. Taken together the data suggests that in BD persistent inflammation drive release of LDN from the bone marrow. The systemic environment influences both LDN and NDN to produce the phenotype described, that in turn drives mucosal inflammation and vascular damage.^1^

## Supplementary Information

**Additional file 1: Supplementary Fig. 1**. LDN (A) and NDN (B) from a healthy individual by cytospins × 20 and × 40.

## Data Availability

Not applicable.

## Footnotes

1 During the preparation of this manuscript, Chen et al. reported on increased LDN in patients with active or vascular Behcet’s disease (doi:10.1136/annrheumdis-2020-219,472) [49].
