# Supplementary figures and images for: Low density neutrophils are increased in patients with Behçet’s disease but do not explain differences in neutrophil function
Source: J Inflamm (Lond). 2022 Mar 31;19:5. doi: 10.1186/s12950-022-00302-1 (PMC8973557; doi:10.1186/s12950-022-00302-1)

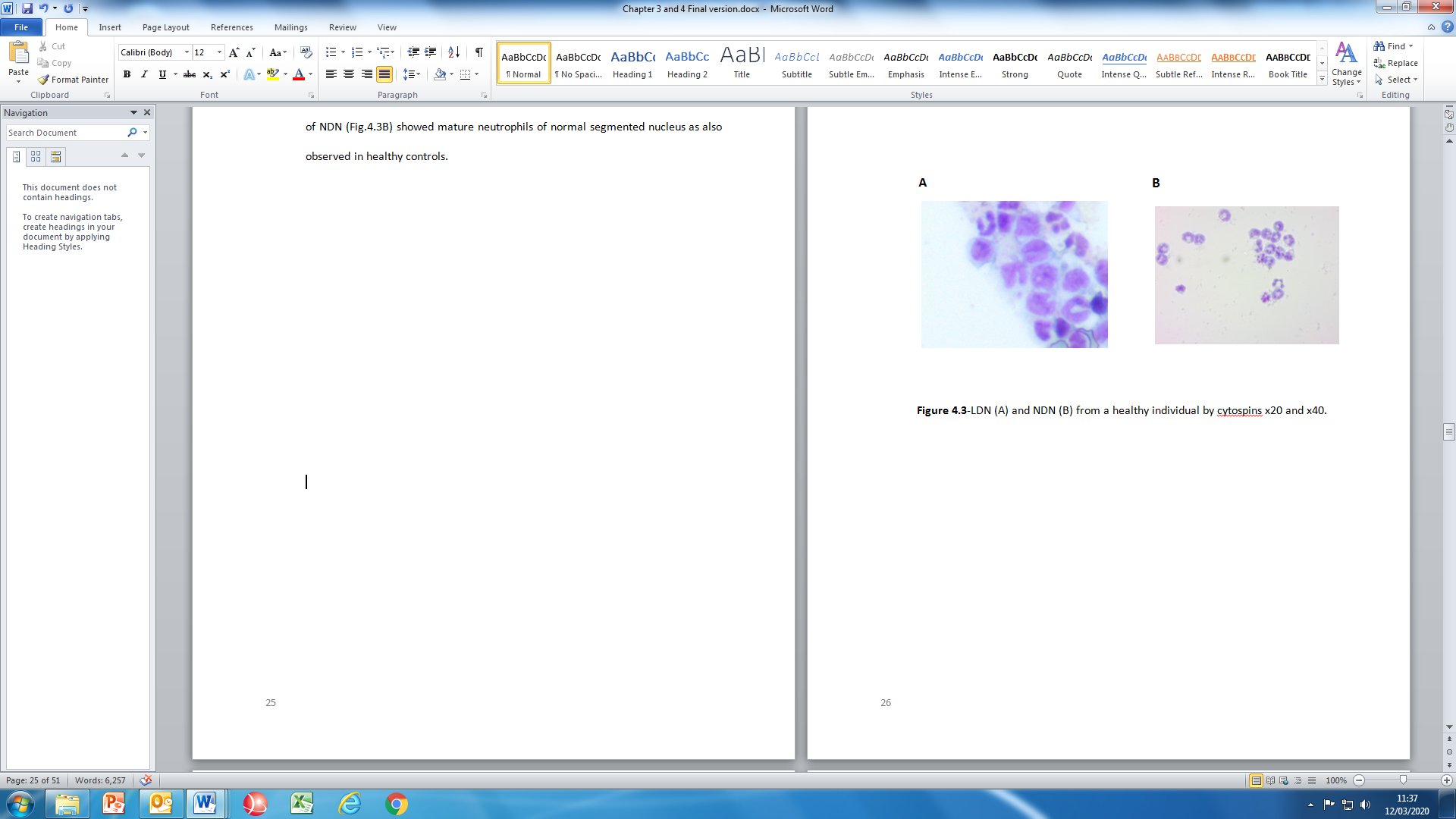


Supplementary Figure 1 -LDN (A) and NDN (B) from a healthy individual by cytospins x20 and x40.

Supplement: Supplementary file 1 — Additional file 1: Supplementary Fig. 1. LDN (A) and NDN (B) from a healthy individual by cytospins × 20 and × 40. [file 12950_2022_302_MOESM1_ESM.docx]
